# An Updated Overview of Existing Cancer Databases and Identified Needs

**DOI:** 10.3390/biology12081152

**Published:** 2023-08-21

**Authors:** Brittany K. Austin, Ali Firooz, Homayoun Valafar, Anna V. Blenda

**Affiliations:** 1 Department of Biomedical Sciences, School of Medicine Greenville, University of South Carolina, Greenville, SC 29605, USA; bkaustin@email.sc.edu; 2 Department of Computer Science and Engineering, College of Engineering and Computing, University of South Carolina, Columbia, SC 29208, USA; ali.firooz@sc.edu; 3 Prisma Health Cancer Institute, Prisma Health, Greenville, SC 29605, USA

**Keywords:** cancer, database, genomic, proteomic, lipidomic, glycomic, clinical trials

## Abstract

**Simple Summary:**

This review examines the current state of cancer databases and identifies key needs in the field. The analysis of 71 databases reveals a lack of dedicated lipidomic and glycomic databases for cancer research, as well as limited proteomic databases. By comparing non-cancer databases, advancements in genomics, proteomics, lipidomics, and glycomics over the past eight years are highlighted. The evaluation of user-friendliness using the FAIRness principle emphasizes the importance of accessibility and usability. Overall, this review emphasizes the growth of cancer databases while identifying areas for improvement, offering valuable insights for researchers, clinicians, and database developers. Addressing these needs will advance cancer research and benefit the wider cancer community.

**Abstract:**

Our search of existing cancer databases aimed to assess the current landscape and identify key needs. We analyzed 71 databases, focusing on genomics, proteomics, lipidomics, and glycomics. We found a lack of cancer-related lipidomic and glycomic databases, indicating a need for further development in these areas. Proteomic databases dedicated to cancer research were also limited. To assess overall progress, we included human non-cancer databases in proteomics, lipidomics, and glycomics for comparison. This provided insights into advancements in these fields over the past eight years. We also analyzed other types of cancer databases, such as clinical trial databases and web servers. Evaluating user-friendliness, we used the FAIRness principle to assess findability, accessibility, interoperability, and reusability. This ensured databases were easily accessible and usable. Our search summary highlights significant growth in cancer databases while identifying gaps and needs. These insights are valuable for researchers, clinicians, and database developers, guiding efforts to enhance accessibility, integration, and usability. Addressing these needs will support advancements in cancer research and benefit the wider cancer community.

## 1. Introduction 

Cancer has been known for a long time, with credible evidence observed in fossilized dinosaurs and human bones from prehistoric times. The earliest record of cancer, written between 1500 and 1600 BC, was discovered in the 19th century [1]. Great physicians and scholars such as Hippocrates, Celsus, and Galen have contributed to a better understanding of cancer, its origin, and nature [1]. The “modern era” of cancer research began in the 19th century and led to the development of the current understanding by several investigators, notably Rudolf Virchow, who stated that cancer is “a disease of cells” [2]. This marked the onset of the war on cancer [3], with physicians and researchers collecting massive amounts of information about the mechanisms of cancer and its influence on genes, proteins, and other biomolecules.

To aggregate this massive amount of information into a central location, databases shared across the international community of researchers are a must. The availability of these databases plays a crucial role in aiding the discovery of the molecular basis of such a complex disease as cancer. The first modern cancer databases emerged in the early 1900s as individual physician’s or institutional projects in the United States or Europe [4]. It was not until 1959 that the American College of Surgeons (ACoS) formally adopted a policy allowing hospital-based cancer registries (i.e., databases) [4], with the primary importance of those databases for “monitoring cancer incidence, mortality, and survival” [5]. Nowadays, the functionality of cancer databases has significantly expanded through the analysis of complex datasets, including genomic, proteomic, glycomic, and clinical trials, to name a few. This review gives an update on the progress of cancer databases development in the last eight years (2015–2023). Periodic review of the existing cancer databases is needed to identify gaps and needs in our existing data collections and analysis tools. This report is one such example, with a focus on surveying the existing databases that aggregate nucleic acids (various forms of RNA and DNA), proteins, carbohydrates, and lipids in the context of cancer.

## 2. Materials and Methods

In this literature review focused on cancer databases in genomics, proteomics, lipidomics, and glycomics, our goal is to analyze their development over the past eight years and identify the existing needs within the cancer research community.

To select the databases for inclusion in the manuscript, we applied two criteria. Firstly, we considered databases published after 2015, as a comprehensive review of the human cancer databases was already available prior to that year [6]. However, we did include a number of papers written before 2015, to illustrate the growth and evolution of certain databases over time. Secondly, we ensured that the selected databases were cancer related. Following these criteria, we compiled a list of 95 databases covering multiple areas of cancer research. From this list, we decided to focus on genomics, proteomics, lipidomics, and glycomics as the fields of interest.

During our analysis, we observed the absence of cancer-related lipidomic and glycomic databases, and only a few cancer-related proteomic databases. Consequently, we decided to incorporate several human non-cancer databases that contain proteomic, lipidomic, and glycomic data. This allowed us to compare the overall progress of knowledge in these fields over the last eight years (2015–2023).

**Figure 1:**
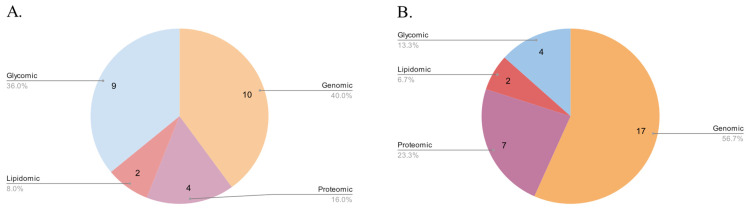
Distribution of Molecular Databases by Type (**A**) Before and (**B**) After 2015.

**Figure 2:**
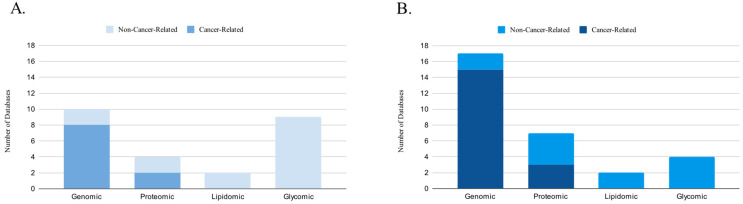
Distribution of Cancer-Related and Non-Cancer-Related Molecular Databases (**A**) Before and (**B**) After 2015.

Furthermore, we examined other types of cancer databases, including databases of cancer clinical trials, web servers, and various other cancer-related databases that did not fit into the aforementioned categories. In total, our final selection comprised 71 databases, consisting of 26 genomic, 10 proteomic, 2 lipidomic, 13 glycomic, 7 dedicated to clinical trials, 6 web servers, and 9 other databases. Out of these, 46 databases were cancer related, while 25 were human non-cancer related. For our analysis, we utilized 108 sources, primarily published after 2015, including 101 original articles and 7 website sources. Additionally, 40 sources were published before 2015, while 61 sources were published after that year (Figure 1 and Figure 2).

Finally, we applied the FAIRness principle to evaluate the user-friendliness of the databases. The FAIR principle emphasizes that databases should be findable, accessible, interoperable, and reusable. To assess these criteria, we conducted our own research on each database. If a database was easily discoverable through web browsers such as Google or Safari, it was considered findable. If the database allowed for login or free access, it was considered accessible. Interoperability was determined by the presence of the database’s own statistical analysis function. Lastly, a database was considered reusable if it provided users with the ability to download data. All the searches and data collection were performed by the human research team, and none of the data collection process relied on ChatGPT or similar tools. The manuscript including all of its tables and figures was generated by the researchers. ChatGPT technology was only used at the last stage of the revision process of the manuscript to check for style, grammar, and spelling.

A database is considered cancer related if its content is predominantly centered around the topic of cancer. These databases often contain specific data related to cancer research, such as genomic data, molecular profiles, clinical information, other cancer-related datasets. For example, “The Cancer Genome Atlas” is a well-known cancer-related database that extensively convers genomic and molecular information specific to various types of cancer. Conversely, a database is classified as non-cancer related if its information is not primarily focused on cancer research. These databases may cover broader scientific topics, such as general protein data or information on various biological processes unrelated to cancer. As an illustration “UniProt” is a non-cancer-related database known for providing comprehensive information on proteins from various organisms, including those not directly related to cancer.

## 3. Results 

### 3.1. Genomic Databases 

Genetic mutations are pivotal in cancer development, and the National Institute of Health (NIH) established the Cancer Genome Atlas (TCGA) to identify significant cancer-causing genomic changes. TCGA has amassed over 11,000 cases spanning 33 tumor types, providing a vast dataset of molecular alterations [7]. Other databases have leveraged TCGA data, such as the OncomiR Cancer Database (OMCD), which utilizes TCGA’s 9500 cancer tissue samples for comparative genomic analyses of miRNA sequencing data [8]. Similarly, Cistrome Cancer serves as a web-based server utilizing TCGA to facilitate data retrieval for integrative gene regulation modeling [9]. Notably, there is a trend of creating smaller user-friendly databases derived from larger ones, exemplified by the cBio Cancer Genomic Portal. Developed to integrate extensive genomic projects, cBio enhances accessibility of raw data to the cancer research community [10].

The International Cancer Genome Consortium (ICGC) is another database aiming to construct a comprehensive catalog of mutational abnormalities observed in major tumor types [11]. ICGC incorporates data from 84 global cancer projects, encompassing approximately 77 million somatic mutations and molecular data from over 20,000 participants [11]. The Human Genome Browser at UCSC acts as a portal for displaying various genomic features, including gene predictions, alignments, polymorphisms, and more [12,13]. The Gene Expression Omnibus Database (GEO), established in 2000, focuses on gene expression and functional genomic datasets, extending beyond genome analysis to genome methylation, chromatin structure, and more [14]. Ensembl, created by Flicek et al. in 2014, provides tools for genomic analysis and has expanded each year. In Ensemble 2018, fields such as gene annotation, comparative genomics, genetics, and epigenomics were added by Zerbino et al. [15,16]. Recently, Martin et al. expanded Ensemble’s genome analysis beyond humans to investigate pangenomes across diverse species in Ensemble 2023 [17].

The National Cancer Institute Genomic Data Commons (GDC) is another prominent cancer database that focuses on storing, analyzing, and sharing genomic and clinical data from cancer patients. The GDC aims to democratize access to cancer genomic data and promote data sharing among researchers. By facilitating the application of precision medicine approaches, the GDC contributes to advancing the diagnosis and treatment of cancer [18,19]. OpenGDC, derived from the GDC, expands upon the existing platform by incorporating the Genomic Data Model. It introduces additional genomic data in Browser Extensible Data (BED) format and provides related metadata in a table-limited key-value format. OpenGDC enhances the efficiency of accessing genomic and clinical data while expanding the amount of information available for analysis [20].

A notable trend observed in cancer databases is the integration of diverse areas of cancer research into a single platform, allowing for the incorporation of multiple functionalities within a unified database. The Gene Expression Omnibus Database (GEO) serves as an example of such integration, offering not only gene expression data but also functional genomic datasets related to genome methylation, chromatin structure, and genome analysis. By encompassing various aspects of cancer research, GEO facilitates comprehensive investigations and analysis within a single database [14].

Futreal et al. emphasize the importance of mutations occurring in more than 1% of genes in the context of human cancers [21]. To facilitate easy access to information about these genes for researchers and physicians, several databases and web servers focus on cataloging them. Examples of such databases include the Network of Cancer Genes [22] and Cancer Hallmark Genes (CHG) [23]. These databases specifically examine genes that are significantly impacted or mutated in cancer.

The Catalogue of Somatic Mutations in Cancer (COSMIC) database is another valuable resource that stores somatic mutation data and related information about human cancer [24]. Since 2004, COSMIC has integrated coding mutations into its database, covering various genetic mechanisms through which somatic mutations contribute to cancer development. These mechanisms include non-coding mutations, gene fusions, copy-number variants, and drug resistance mutations [25]. Additionally, the COSMIC website provides users with the ability to visualize the 3D structure of proteins [25].

Mutagene is a database that delves into the mutational profiles of 37 distinct cancer types. It investigates the underlying components and signatures across over 9000 genomes and exomes, enabling comparisons of mutagenic processes between different types of cancers [26]. The Progenetix project, initiated in 2001, focuses on individual cancer copy number abnormalities (CNAs) profiles and associated metadata. Over the years, the project has expanded its collection of copy number variations (CNVs) and increased the number of samples, resulting in an improved database with enhanced data quality [27,28]. The MutEx database is dedicated to gathering information on the connections between somatic mutations, gene expression, and patient survival rates [29].

Oncomine is a cancer microarray database that conducts genome-wide expression analyses to identify tumor-related genes, novel biomarkers, and therapeutic targets [30]. Oncomine 3.0, developed in 2007, serves the biomedical research community by collecting, standardizing, analyzing, and delivering cancer transcriptome data [31]. Rhodes et al. utilized the Oncomine 3.0 database to identify genes, pathways, cancer types, and subtypes [31]. Currently, Oncomine has focused its efforts on assay analysis to assist oncologists in making clinical decisions. Their latest functional version is Oncomine Comprehensive Assay v3 (OCAv3), which covers 151 cancer-associated genes, allowing the detection of single nucleotide variants (SNVs), multiple-nucleotide variants (MSVs), and small insertions/deletions (indels) [32]. Since 2017, OCAv3 has been used in clinical settings to support oncologists in determining therapeutic courses. Additionally, Oncomine has developed Oncomine Comprehensive Assay Plus (OCA-Plus), which covers 501 genes, with 144 genes overlapping with OCAv3. OCA-Plus includes assays for microsatellite instability (MSI) and tumor mutational burden (TMB), all in one workflow. Currently, the update of OCA-Plus is under development before its release into clinical settings [32].

#### Cancer Specific Databases

Lung Cancer Explorer (LCE) is a database specifically dedicated to lung cancer. It enables researchers and clinicians to explore lung cancer data and perform various analyses [33]. PROMISE (Prostate Cancer Precision Medicine Multi-Institutional Collaborative Effort) is a consortium that aims to establish a collection of de-identified clinical and genomic patient data linked to patient outcomes. PROMISE involves different committees focusing on genomic data, statistical analyses, patient advocacy, and other aspects to advance precision medicine in prostate cancer research [34].

HCCDB is a notable database that focuses on hepatocellular carcinoma (HCC), a type of liver cancer. It serves as an online resource providing a consolidated platform for researching gene expression in relation to HCC. HCCDB allows for different types of analyses, including tissue-specific and tumor-specific expression analysis, as well as co-expression analysis [35].

OncoReveal database specifically focuses on non-small cell lung cancer (NSCLC) and colorectal cancer (CRC). It provides a platform for researchers and clinicians to access relevant data and insights related to these specific cancer types. For a summary of all the GENOMIC databases and web servers reviewed, as well as a visual representation of the information, please refer to Figure 1 and Table 1. 

### 3.2. Proteomic Databases

The Clinical Proteomic Tumor Analysis Consortium (CPTAC) is a database created by the National Cancer Institute (NCI) that analyzes cancer biospecimens using mass spectrometry. It identifies and characterizes protein alterations within tumor samples, providing this proteomic data to the public in an accessible manner. CPTAC collaborates with the Cancer Genome Atlas (TCGA) to provide proteomic input for breast, colorectal, and ovarian tissue samples within the TCGA framework [36,37]. Lindgren’s paper in 2021 discusses the data application programming interface (API) created by CPTAC, which distributes processed datasets in a consistent format, facilitating advanced analysis [38].

The String database integrates known and predicted associations between proteins, including physical interactions and functional associations. It utilizes text mining, pathway analysis, and interaction databases to consolidate knowledge on protein interactions [39].

The UALCAN web portal, established in 2017, allows the cancer community to analyze and access cancer transcriptome, proteomics, and patient survival data. It has been expanded to include microRNAs, long non-coding RNAs (lncRNAs), DNA methylation data, and proteomics from CPTAC [40].

CanProVar focuses on human cancer proteome variations, providing a platform for the storage and retrieval of single amino acid alterations observed in cancer. Researchers can efficiently query and explore these alterations using CanProVar, which offers easy accessibility and search capabilities based on gene or protein IDs, cancer types, chromosome locations, and pathways. CanProVar 2.0 is the latest version, featuring a tenfold increase in the number of variations and improved search functionality [41].

The following resources mentioned below are not specifically cancer related, but they contribute to the understanding of proteomics and its role in cancer research. The RCSB Protein Data Bank provides access to 3D structures of biological macromolecules, aiding in the comprehension of protein and macromolecule structures [42]. The Universal Protein Resource (UniProt) is an open-source repository of protein sequences and functional annotations, offering visualizations of protein subcellular localization, structure, and interactions [43,44]. Proteome Discoverer is a data software used to convert mass spectrometry files to protein identifications [45]. SWISS-PROT and TrEMBL are protein sequence databases that provide information on protein functions, domains, structures, and post-translational modifications [46]. jPOST is a proteomic database that allows users to observe the frequency of post-translational modification detection, examine the co-occurrence of phosphorylation sites, and explore peptide sharing among proteoforms [47]. MatrisomeDB is a selected proteomic database containing data from various extracellular matrix (ECM) studies, offering a searchable repository of useful information related to normal tissues, cancers, and disorders [48]. Table 2 provides a summary of the mentioned proteomic databases.

### 3.3. Lipidomic Databases

Lipidomics plays an increasingly important role in cancer research due to the involvement of lipids in cancer growth, including their role in membrane structure, energy storage, and signal transduction. Some cancer cells, such as breast and ovarian cancer cells, rely on fatty acid oxidation for energy, while lipid accumulation has been observed in certain cancer cells [49]. Understanding the specific lipids affected in different types of cancer can aid in the development of improved treatments and diagnostic approaches. 

Although lipidomics in cancer research is still under development, studies have explored the role of lipids in various cancers. For example, a study on lipidomics in colorectal cancer suggested that lipids may play a role in cancer development. However, further research involving larger populations and different cancer stages is needed. Additionally, investigating other factors contributing to increased lipid production in cancer cells is recommended [50]. 

While there is currently no cancer-specific lipidomic database, there are non-cancer lipidomic databases that provide valuable resources (Table 3). One such database is DBLiPro, which aims to establish a comprehensive knowledge base of human lipid metabolism and offers lipidome-centric analysis tools [51]. Lipid Maps is another notable database, consisting of two components: the Lipid Maps Proteome database (LMPD), which focuses on proteins [52], and the Lipid Maps Structure database (LMSD), which provides information on lipid structures and annotations of biologically relevant lipids [53]. In 2020, Lipid Maps updated its classification system and shorthand notation for lipid structures, including categories such as fatty acyls and glycerolipids [54].

### 3.4. Glycomic Databases

Galectin studies and glycomic research have gained importance in cancer studies due to involvement in crucial processes such as angiogenesis, metastasis, cell division, and immune evasion. Specific galectins and glycans play significant roles in these processes, modifying immune cells through interactions with glycosylated proteins and lipids. Understanding the effects of galectins and glycans and their alterations in cancer can lead to improved diagnostics and treatment. Changes in galectin expression may be influenced by protein trafficking and alterations in the glycocalyx composition of cancer cells [55,56,57,58].

While most glycomic databases are not cancer-specific, they provide valuable insights into glycan structure, function, and the field of glycoproteomics. Glycoproteomics focuses on identifying, locating, characterizing, and studying the abundance and role of glycosylated proteins in biological processes, including cancer. Mass spectrometry is commonly used for studying glycan alterations in cancer [59,60,61,62,63].

Given the limited number of cancer-related glycomic databases, incorporating glycomic information into cancer-related databases is crucial. Key glycomic databases include UniCarb-DB, UniPep, GlycoGene database (GGDB), and Glycome-DB. These databases offer a wealth of glycan and glycoproteomics data, enabling the examination of glycan structures, fragment data, biological context, and more [64,65,66,67,68,69,70]. Recent advancements in the field include GlycoRDF, GRITs database, GlyTouCan, Lectin Frontier Database (LfDB), and Carbohydrate Structure Database (CSDB), aiming to improve data quality, coverage, and standardization of carbohydrate notations [71,72,73,74,75,76], (Table 4).

### 3.5. Clinical Trial Databases 

Clinical trials play a crucial role in cancer research, as they help evaluate the safety and effectiveness of diagnostics, treatments, and medication development. Integrating clinical trial databases is essential for understanding the impact of trials and patient demographics on the development of improved and personalized treatments. Here are several clinical trial databases relevant to cancer research: (1) Clinical Genomic Database (CGD): CGD provides a comprehensive collection of genetic conditions where genetic information can influence appropriate supportive care, medical decision-making, prognostic assessments, reproductive choices, and help avoid unnecessary diagnostic testing [77]. (2) Foundation Medicine Adult Cancer-Clinical Dataset: This dataset serves as a valuable resource for researching uncommon mutations and disorders, verifying their clinical importance, and discovering novel treatment options [78]. (3) Curated Cancer Clinical Outcomes Database (C3OD): C3OD integrates electronic medical records, tumor registry, biospecimen, and data registry to facilitate easier access to patient data in a unified location. Its goal is to accelerate eligibility screening for research purposes [79]. (4) Danish Head and Neck Cancer Database: Started in the early 1960s, this database focuses on a national strategy for multidisciplinary treatment of head and neck cancer in Denmark. It is utilized to describe the effects of reduced waiting time, changing epidemiology, and the influence of comorbidity and socioeconomic factors [80]. (5) National Cancer Database (NCDB): Over the past three decades, NCDB has evolved significantly, aggregating and categorizing approximately 40 million patient records from over 1500 hospitals. Its aim is to enhance the quality of cancer patient care [81]. (6) Surveillance, Epidemiology, and End Results (SEER) database: SEER focuses on investigating the history of colorectal cancer and patient care, providing valuable insights to the field [82]. (7) ClinVar: ClinVar is a public database designed for clinical laboratories, researchers, and expert panels. Launched in 2013, it contains over 600,000 submitted records from 1000 submitters, representing 430,000 unique variants. ClinVar enables data comparison among researchers [83]. 

Table 5 includes more detailed information about each database, its main features and scope.

### 3.6. Other Cancer Databases

Several other databases are also important for cancer research. The Database of Epigenetics Modifier (dbEM) contains potential targets for cancer treatment and information on mutations, copy number variations, and gene expression in tumor samples [84]. The Cancer Research Database (CRDB) explores the correlation between cancer and the COVID-19 pandemic, scoring other databases based on cancer types, sample size, omics results, and user interface [85]. The Comprehensive Review of Web Servers and Bioinformatics Tools for Cancer Prognosis Analysis discusses databases that examine prognostic biomarkers and survival rates, including PROGgene V2 [86,87]. The Cancer Drug Resistance (CancerDR) database provides information on anti-cancer drugs and their profiling across cancer cell lines [88]. DriverDB identifies driver genes/mutations using algorithms [89], while LncRNA2Target 2.0 and Lnc2Cancer focus on long non-coding RNAs associated with cancer [90,91]. The Genotype-Tissue Expression (GTEx) database investigates the relationship between genetic variation and gene expression in humans [92]. These evolving databases additionally contribute to improved diagnosis, prognosis, and therapeutic interventions in cancer research (Table 6).

Additionally, there are other databases that are non-cancer related that are being used alongside cancer databases to help increase the data surrounding the studied topic. Examples of these databases are The Comparative Toxicogenomic Database (CTD) connects toxicological data related to chemicals, genes, phenotypes, diseases, and exposures to enhance our understanding of human health [93]. The Therapeutic Target Database (TTD) provides information on known therapeutic proteins and nucleic acid targets. It includes pathway information and details about drugs/ligands directed at each target. The database offers sequences, 3D structures, functions, nomenclature, drug/ligand binding properties, drug usage, and effects associated with each target. Over time, TTD has expanded its repository to include target-regulating microRNAs, transcription factors, target-interacting proteins, as well as patented agents and their corresponding targets [94,95]. The Pharmacogenomics Knowledge Base (PharmGKB) presents genotypes, molecular data, and clinical information in a pathway-oriented representation. It also provides Very Important Pharmacogenes (VIP) summaries and links to additional external sources for further exploration. As of April 2021, PharmGKB contained annotated data for 715 drugs, 1761 genes, 227 diseases, and 165 clinical guidelines and drug labels [96,97]. DrugBank is a database that offers detailed molecular information about medications, including mechanisms, interactions, and targets. The most recent edition is DrugBank 5.0 [98]. These databases also are being used alongside cancer-related databases such as Ualcan, (protein database), the Cancer Research Database, and CancerResource, which is now a retired database. 

#### Retired Databases

Development of databases has seen a number of changes, with some databases being retired while new ones emerge to fill the gaps. One retired database is the CancerResource database. This database was a comprehensive cancer-related data repository that integrated information from multiple databases to provide a fuller and more interactive resource. One key aspect of CancerResource was its focus on understanding how medications or drug-related substances interact with specific genes or proteins [99]. To achieve its comprehensive approach, CancerResource utilized several databases, including the Comparative Toxicogenomic Database (CTD), Therapeutic Target Database (TTD), Pharmacogenomics Knowledge Base (PharmGKB), and DrugBank. In the last eight years, the CancerResource database has expanded, encompassing approximately 91,000 drug-target relations, over 2000 cancer cell lines, and drug sensitivity data for about 50,000 drugs. CancerResource also allowed users to upload external expression and mutation data, enabling comparison with the database’s cell lines [100]. It is worth noting that as individual databases grow, interconnected databases such as CancerResource benefit from the acquisition of new and valuable information.

Genomic databases have also experienced the retiring of some of their databases. Among these retired genomic databases are the Roche Genomic Cancer Database and the Cancer Genes database [101,102], both of which played crucial roles in studying mutations surrounding cancer genes. The Roche Cancer Genome Database 2.0 (RCGDB) served as a comprehensive platform that combined different human mutation databases into a single location. This database offered interactive search capabilities for genes, samples, cell lines, diseases, and pathways, providing users with a centralized resource for accessing and analyzing cancer-related information. RCGDB also allowed for customized searches based on specific filter criteria, enabling researchers to address regularly occurring queries efficiently [103]. 

The contributions of the retired databases to cancer research have been significant, and their retirement leaves an opportunity for new advancements in the fields. 

Similarly, the glycomic research community has experienced a transition in databases. While some database such as the GlycoSuite Database, EuroCarb, GlycoBase, and GlycoStore [64,66,70,71] have been retired and become inaccessible on web browsers such as Google Chrome and Safari, it is essential to acknowledge the wealth of information they previously provided. These databases were valuable resources for researchers, clinicians, and healthcare professionals studying glycomic data and its implications in various disease and biological processes.

### 3.7. Web-Based Servers 

Web servers are instrumental in cancer research, offering various functionalities and benefits. GSCALite, for example, performs comprehensive analysis of cancer-related genes, including differential expression, survival analysis, genomic variation assessment, cancer pathway activity, miRNA regulation, drug sensitivity, and normal tissue expression [103]. OMIM serves as an online catalog, providing extensive information on genetic phenotypes, DNA/protein sequences, references, and mutational databases [104]. GEPIA is a web-based tool that enables interactive analysis of differential gene expression, correlation, survival, gene similarity, and dimensional reduction [105]. PepQuery facilitates proteomic validation of genomic alterations through simulations and experimental data [106]. These web servers play a critical role in empowering researchers and enabling in-depth exploration and analysis of cancer data (Table 7).

## 4. Discussion

Databases have undergone significant growth and development in the past eight years, manifesting in various ways. Firstly, databases have expanded their information by continually adding more data. For instance, CanProVar 2.0 has experienced a tenfold increase in its content since its inception, enabling the dissemination of more comprehensive information. The sharing of data has emerged as a crucial focus for glycomic researchers, leading to the creation of databases such as GlyTouCan and the Carbohydrate Structure database. These databases aim to address integration challenges and other issues prevalent in glycan databases. CancerResource is another exemplar of databases sharing information, as it derives data from multiple sources.

Furthermore, databases have broadened their research scope by incorporating additional topics beyond their original areas of focus. A notable instance is Ualcan, a proteomic database that integrated microRNA and lncRNA data to explore patient survival outcomes. This expansion reflects the inclination of databases to explore diverse research domains within a single platform.

The second aspect of database growth pertains to database design and usability. Database developers and curators have striven to enhance user-friendliness, often evaluated through the FAIRness principle. This principle encompasses various criteria, including findability, accessibility, interpretability, and reusability, to determine the fairness and usability of scientific research, including databases [107]. A user-friendly database should be discoverable, easily accessible, interpretable, and allow data reuse for any purpose. Many databases examined in this study have endeavored to improve user-friendliness through website redesign, resulting in enhanced search engines and capabilities such as copying/pasting or downloading datasets. Additionally, efforts have been made to enable users to create their datasets within the database.

**Figure 3:**
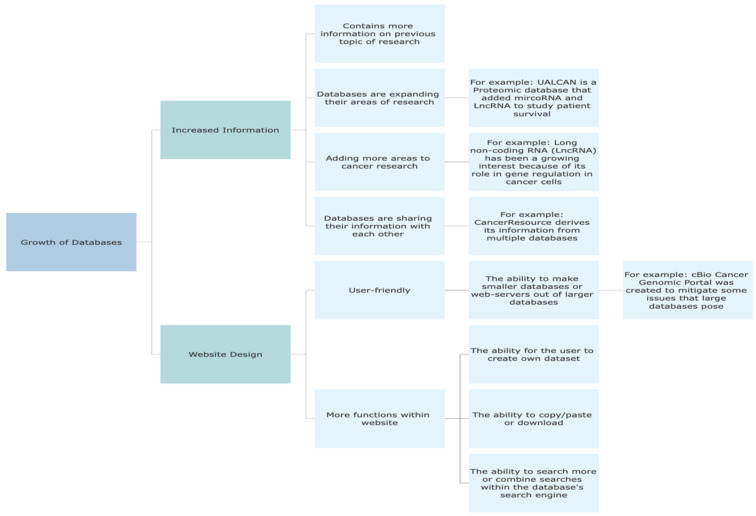
Emerging Trends in Database Development Over the Last Eight Years.

Overall, databases have experienced growth in terms of data expansion and user-friendly design. These advancements facilitate information sharing, enable broader research exploration, and contribute to the usability and accessibility of scientific research databases (Figure 3).

## 5. Conclusions

In conclusion, our search summary of existing cancer databases reveals significant growth and development over the past eight years. We have identified the need for more cancer-related lipidomic and glycomic databases, as well as the scarcity of proteomic databases in the cancer domain. Additionally, we have highlighted the importance of user-friendliness in database design and adherence to the FAIRness principles. This comprehensive analysis provides valuable insights into the current state of cancer databases and the areas that require further attention and improvement.

## Figures and Tables

**Table 1:** Genomic Databases.

Name	Content/Functionality	Web Service	Downloadable	Analytics	Fairness	Website
The Cancer Genome Atlas Cases = 11,315	Genome sequencing across 33 tumor types	Yes	Yes	Yes	F, A, I, R	https://www.cancer.gov/ccg/research/genome-sequencing/tcga (accessed on 14 March 2023)
OncomiR Cancer Database OMCD Cases = 9500	Comparative genomic analysis of miRNA data sequencing	Yes	N/A	Yes	F, A, I	http://www.oncomir.org/cgi-bin/dbSearch.cgi (accessed on 14 March 2023)
cBio Cancer Genomic Portal	Genomic analysis of cancer-related genes	Yes	Yes	Yes	F, A, I, R	https://www.cbioportal.org/ (accessed on 14 March 2023)
International Cancer Genome Consortium (ICGC) Donors ~24,500	Catalog of mutational abnormalities in major tumor types	Yes	Yes	Yes	F, A, I, R	https://dcc.icgc.org/ (accessed on 14 March 2023)
Human Genome Browser at USCS	Genomic data	Yes	Yes		F, A, R	https://genome.ucsc.edu/index.html (accessed on 14 March 2023)
Gene Expression Omnibus Database (GEO)	Gene expression data	Yes	Yes		F, A, R	https://www.ncbi.nlm.nih.gov/geo/ (accessed on 14 March 2023)
Ensembl	Genomic analysis	Yes	Yes		F, A, R	https://www.ensembl.org/index.html (accessed on 14 March 2023)
National Cancer Institute Genomic Commons (GDC) Cases = 22,000	Storage, analysis, and sharing of clinical data of patients	Yes	Yes	Yes	F, A, I, R	https://portal.gdc.cancer.gov/ (accessed on 14 March 2023)
Network of Cancer Genes	Cancer genes, healthy drivers and their properties	Yes	Yes	Yes	F, A, I, R	http://ncg.kcl.ac.uk/index.php (accessed on 14 March 2023)
Catalogue of Somatic Mutation in Cancer (COSMIC)	Somatic mutations in human cancer	Yes	Yes	Yes	F, A, I, R	https://cancer.sanger.ac.uk/cosmic (accessed on 14 March 2023)
Mutagene	Mutational profiles in 37 cancer types	Yes	Yes	Yes	F, A, I, R	https://www.ncbi.nlm.nih.gov/research/mutagene/ (accessed on 14 March 2023)
Progenetix Samples = 142,063	Cancer copy number abnormalities (CNA)	Yes	Yes	Yes	F, A, I, R	https://progenetix.org/ (accessed on 14 March 2023)
MutEx	Stores and explores the relationships between gene expression, somatic mutation,mutational burden and and survival	Yes	Yes	Yes	F, A, I, R	http://www.innovebioinfo.com/Databases/Mutationdb_About.php (accessed on 14 March 2023)
Oncomine	Precision oncology through next-generation sequencing	Yes	Yes	Yes	F, A, I, R	https://www.oncomine.com/ (accessed on 14 March 2023)
Lung Cancer Explorer (LCE) Entries = 356	Enables the exploration of lung cancer in various analyses	Yes	Yes		F, A, R	https://lce.biohpc.swmed.edu/lungcancer/imageset_tcga.php (accessed on 14 March 2023)
Prostate Cancer Precision Medicine Multi-Institutional Collaborative Effort PROMISE	Analyzes prostate cancer genes and patient outcomes	Yes		Yes	F, A, I	https://www.prostatecancerpromise.org/research/ (accessed on 14 March 2023)
HCCDb	Information about hepatocellular carcinoma	Yes	Yes		F, A, R	http://lifeome.net/database/hccdb/home.html (accessed on 14 March 2023)

**Table 2:** Proteomic Databases.

Name	Content/Functionality	Web Service	Downloadable	Analytics	Fairness	Website
Clinical Proteomic Tumor Analysis Consortium (CPTAC)	Application of large-scale proteome and genome analysis	Yes	Yes	Yes	F, A, I, R	https://proteomics.cancer.gov/programs/cptac (accessed on 14 March 2023)
String Database	Protein-protein interactions, functional enrichment analysis	Yes	Yes	Yes	F, A, I, R	https://string-db.org/ (accessed on 14 March 2023)
Ualcan	Analyzes cancer transcriptome, proteome, and patient survival data	Yes	N/A	Yes	F, A, I	https://ualcan.path.uab.edu/ (accessed on 14 March 2023)
CanProVar	Proteomic variations	Yes	Yes		F, A, R	http://119.3.70.71/CanProVar/index.html (accessed on 14 March 2023)
RCSB Protein Data Bank *	Works with UniProt and analyzes protein structures	Yes	Yes	Yes	F, A, I, R	https://www.rcsb.org/ (accessed on 14 March 2023)
Universal Protein Resource (UniProt) *	Information about protein structures and interactions	Yes	Yes	Yes	F, A, I, R	https://www.uniprot.org/ (accessed on 14 March 2023)
Proteome Discover *	Not free to access (attempted to access on 14 March 2023)					
Swiss-Prot and TrEMBL *	A part of the UniProt database	Yes	Yes	Yes	F, A, I, R	https://www.uniprot.org/uniprotkb (accessed on 14 March 2023)
jPOST *	Post-translational modifications on proteins	Yes	Yes	Yes	F, A, I, R	https://globe.jpostdb.org/ (accessed on 14 March 2023)
MatrisomeDB *	Proteomic data from studies of ECM **	Yes	Yes		F, A, R	https://matrisomedb.org/ (accessed on 14 March 2023)

* Databases not related to cancer. ** ECM, extracellular matrix.

**Table 3:** Lipidomic Databases.

Databases	Content/Functionality	Web Service	Downloadable	Analytics	Fairness	Website
DBLiPro *	Focuses on human lipid metabolism and provides lipidome-centric analysis	Yes	Yes	Yes	F, A, I, R	http://lipid.cloudna.cn/home (accessed on 14 March 2023)
Lipid Maps *	Lipid structures	Yes	Yes	Yes	F, A, I, R	https://www.lipidmaps.org/ (accessed on 14 March 2023)

* Databases not related to cancer.

**Table 4:** Glycomic Databases.

Name	Content/Functionality	Web Service	Downloadable	Analytics	Fairness	Website
UniCarb-DB *	Carbohydrates characterized by LC-MS	Yes	Yes	Yes	F, A, I, R	https://unicarb-db.expasy.org/about (accessed on 14 March 2023)
UniPep *	N-linked glycosites for proteomic analyses	Yes	Yes		F, A, R	https://unipep.systemsbiology.net/ (accessed on 14 March 2023)
GlycoGene (GGDB) *	Information about glycogenes	Yes	Yes		F, A, R	https://www.glycogene.com/ (accessed on 14 March 2023)
Glycome-DB *	A part of the GlyTouCan database					http://www.glycome-db.org/ (accessed on 14 March 2023)
GlycoRDF *	Glycomics data	Yes	Yes		F, A, R	https://github.com/glycoinfo/GlycoRDF/wiki (accessed on 14 March 2023)
GRITs Toolbox*	Processing, annotating and archiving of glycomics data with a focus on MS data	Yes	Yes	Yes	F, A, I, R	http://www.grits-toolbox.org/ (accessed on 14 March 2023)
GlyTouCan *	Glycan structure repository	Yes	Yes		F, A, R	https://glytoucan.org/ (accessed on 14 March 2023)
The Lectin Frontier Database (LfDB) *	Lectin-standard oligosaccharide interactions	Yes			F, A	https://acgg.asia/lfdb2/ (accessed on 14 March 2023)
Carbohydrate Structure Database (CSDB) *	Manually curated natural carbohydrate structures, taxonomy, bibliography, NMR, and other data	Yes		Yes, this is done by an external source	F, A, I, R	http://csdb.glycoscience.ru/database/index.html (accessed on 14 March 2023)

* Databases not related to cancer.

**Table 5:** Clinical Trial Databases.

Name	Content/Functionality	Web Service	Downloadable	Analytics	Fairness	Website
Clinical Genomic Database (GCD)	Genetic information pertaining to patient care	Yes	Yes		F, A, R	https://research.nhgri.nih.gov/CGD/ (accessed on 14 March 2023)
Foundation Medicine Adult-Cancer-Clinical Dataset	Clinical relevance of rare alterations and diseases	Yes	Yes	Yes	F, A, I, R	https://gdc.cancer.gov/about-gdc/contributed-genomic-data-cancer-research/foundation-medicine/foundation-medicine (accessed on 14 March 2023)
A Curated Cancer Clinical Outcome Database (C3OD)	Cannot access (14 March 2023)					
Danish Head and Neck Cancer Database	National guidelines, clinical studies for improved treatment	Yes	Yes		F, A, R	https://www.dahanca.dk/IndexPage (accessed on 14 March 2023)
National Cancer Database (NCDB)	Requires login access				F	https://www.facs.org/quality-programs/cancer-programs/national-cancer-database/ (accessed on 14 March 2023)
Surveillance Epidemiology and End Results (SEER) *	Information on cancer statistics	Yes			F, A	https://seer.cancer.gov/ (accessed on 14 March 2023)
ClinVar *	Information about genomic variations and their relationship to human health.	Yes	Yes		F, A, R	https://www.ncbi.nlm.nih.gov/clinvar/ (accessed on 14 March 2023)

* Databases not related to cancer.

**Table 6:** Other Databases.

Name	Content/Functionality	Web Service	Downloadable	Analytics	Fairness	Website
Database of Epigenetics Modifiers (dbEM) *	Genomic information about epigenetic modifiers from cancerous and normal genomes	Yes			F, A	https://webs.iiitd.edu.in/raghava/dbem/index.php (accessed on 14 March 2023)
Cancer Research Database (CRDB)	Analyses of other cancer research databases	Yes			F, A	https://www.habdsk.org/crdb (accessed on 14 March 2023)
PROGgene	Pan Cancer Prognostics	Yes		Yes	F, A, I	http://www.progtools.net/gene/index.php (accessed on 14 March 2023)
Cancer Drug Resistance (CancerDR)	Information about anticancer drugs and their effectiveness against cancer cell lines	Yes	Yes		F, A, R	https://webs.iiitd.edu.in/raghava/cancerdr/index.html (accessed on 14 March 2023)
DriverDBv3	Cancer driver genes and mutations	Yes	Yes	Yes	F, A, I, R	http://driverdb.tms.cmu.edu.tw/ (accessed on 14 March 2023)
LncRNA2Target 2.0 *	Server unreachable (14 March 2023)					
Lnc2Cancers 3.0	Server unreachable (14 March 2023)					
Genotype Expression Project (GTEx) *	Gene expression data, QTLs, and histology images	Yes	Yes		F, A, R	https://www.gtexportal.org/home/ (accessed on 14 March 2023)
Comparative Toxicogenomic Database (CTD) *	Chemical–gene/protein interactions, chemical–disease and gene–disease relationships	Yes	Yes	Yes	F, A, I, R	http://ctdbase.org/ (accessed on 14 March 2023)
Therapeutic Target Database (TTD)	Therapeutic protein and nucleic acid targets, the targeted disease, pathway information and the corresponding drugs	Yes	Yes	Yes	F, A, I, R	https://db.idrblab.net/ttd/ (accessed on 14 March 2023)
Pharmacogenomics Knowledge Base (PharmGKB)	Knowledge about the impact of genetic variation on drug response	Yes	Yes		F, A, I, R	https://www.pharmgkb.org/ (accessed on 14 March 2023)
DrugBank	Information about drugs, mechanisms of action, and interactions	Yes	Yes		F, A, I, R	https://go.drugbank.com/ (accessed on 14 March 2023)

* Databases not related to cancer.

**Table 7:** Web Servers.

Name	Content/Functionality	Web Service	Downloadable	Analytics	Fairness	Website
Gene Set Cancer Analysis (GSCALite)	Analysis platform for gene set cancer analysis	Yes			F, A	http://bioinfo.life.hust.edu.cn/web/GSCALite/ (accessed on 14 March 2023)
Online Mendelian Inheritance in Man (OMIM)	Online catalog of human genes and genetic disorders	Yes	Yes		F, A, R	https://www.omim.org/ (accessed on 14 March 2023)
Gene Expression Profiling Interactive Analysis (GEPIA) *	Analysis of RNA sequencing expression data	Yes	Yes	Yes	F, A, I, R	http://gepia.cancer-pku.cn/ (accessed on 14 March 2023)
PepQuery *	Universal targeted peptide search engine	Yes	Yes		F, A, R	http://www.pepquery.org/ (accessed on 14 March 2023)

* Web servers not related to cancer.

## Data Availability

All data are available in the manuscript.
